# The Influence of Copy-Number of Targeted Extrachromosomal Genetic Elements on the Outcome of CRISPR-Cas Defense

**DOI:** 10.3389/fmolb.2016.00045

**Published:** 2016-08-31

**Authors:** Konstantin Severinov, Iaroslav Ispolatov, Ekaterina Semenova

**Affiliations:** ^1^Skolkovo Institute of Science and TechnologySkolkovo, Russia; ^2^Waksman Institute of Microbiology, Rutgers, The State University of New JerseyPiscataway, NJ, USA; ^3^Institute of Molecular Genetics, Russian Academy of SciencesMoscow, Russia; ^4^Department of Physics, University of Santiago de ChileSantiago, Chile

**Keywords:** CRISPR-Cas interference, CRISPR-Cas adaptation, plasmid maintenance, bacteriophage infection, primed adaptation

## Abstract

Prokaryotic type I CRISPR-Cas systems respond to the presence of mobile genetic elements such as plasmids and phages in two different ways. CRISPR interference efficiently destroys foreign DNA harboring protospacers fully matching CRISPR RNA spacers. In contrast, even a single mismatch between a spacer and a protospacer can render CRISPR interference ineffective but causes primed adaptation—efficient and specific acquisition of additional spacers from foreign DNA into the CRISPR array of the host. It has been proposed that the interference and primed adaptation pathways are mediated by structurally different complexes formed by the effector Cascade complex on matching and mismatched protospacers. Here, we present experimental evidence and present a simple mathematical model that shows that when plasmid copy number maintenance/phage genome replication is taken into account, the two apparently different outcomes of the CRISPR-Cas response can be accounted for by just one kind of effector complex on both targets. The results underscore the importance of consideration of targeted genome biology when considering consequences of CRISPR-Cas systems action.

CRISPR (Clustered Regularly Interspaced Short Palindromic Repeats)—Cas (*C*RISPR *as*sociated proteins) systems provide their prokaryotic hosts with adaptive small-RNA-based immunity against mobile genetic elements such as viruses and plasmids (Barrangou et al., 2007; Brouns et al., 2008; Marraffini and Sontheimer, 2008). While evolutionary and mechanistically diverse, all CRISPR-Cas systems comprise (i) arrays of DNA repeats separated by unique spacers and (ii) *cas* genes (Makarova et al., 2015). Functionally, CRISPR-Cas systems can be divided into two modules. The acquisition module appropriates spacers from foreign DNA into CRISPR arrays and consists of Cas1 and Cas2, which function as stand-alone proteins or as fusions to other Cas proteins. Homologs of Cas1 and Cas2 are present in most functional CRISPR-Cas systems (Makarova et al., 2015). The Cas1 and Cas2 proteins from *Escherichia coli*, alone, are able to perform the spacer acquisition reaction *in vitro* (Nunez et al., 2015), and are also sufficient *in vivo* in the absence of other Cas proteins to incorporate new spacers into a minimal CRISPR array consisting of a single repeat and short upstream leader sequence (Yosef et al., 2012; Arslan et al., 2014). When a spacer is acquired, a new copy of CRISPR repeat is also generated (Barrangou et al., 2007; Yosef et al., 2012; Arslan et al., 2014). Spacer acquisition catalyzed by Cas1 and Cas2 only is referred to as “naïve CRISPR adaptation” (Datsenko et al., 2012; Fineran and Charpentier, 2012). Acquired spacers become a source of small CRISPR RNAs (crRNAs) programmed against DNA from which they originated. Individual crRNAs are bound to Cas proteins from the interference module and the resulting “effector complex” recognizes foreign nucleic acids through complementary interactions between the targeted sequence (protospacer) and matching crRNA spacer. Unlike Cas1 and Cas2, the interference module proteins are highly diverse and this diversity forms a basis for classification of CRISPR-Cas systems into several types (Makarova et al., 2015). In DNA targeting Type I, Type II, and Type V CRISPR-Cas systems protospacer recognition requires, in addition to crRNA with complementary spacer, a protospacer adjacent motif (PAM) (Deveau et al., 2008; Mojica et al., 2009; Shmakov et al., 2015; Zetsche et al., 2015) recognized by interference module proteins (Sashital et al., 2012; Anders et al., 2014; Hayes et al., 2016). Upon target DNA recognition, a stable R-loop complex containing locally melted protospacer DNA and an RNA-DNA heteroduplex is formed (Jore et al., 2011; Szczelkun et al., 2014). R-loop formation is followed by target DNA destruction either by single-peptide effectors of Type II and V (Sapranauskas et al., 2011; Shmakov et al., 2015; Zetsche et al., 2015) or through recruitment of additional “executor” endonuclease (Cas3) in Type I systems (Sinkunas et al., 2011; Gong et al., 2014; Hochstrasser et al., 2014; Huo et al., 2014). Cas1 and Cas2 are not required for interference either *in vivo* (Brouns et al., 2008) or *in vitro* (Westra et al., 2012; Mulepati and Bailey, 2013).

Point mutations in protospacer or PAM decrease effector complex affinity (Semenova et al., 2011). *In vitro*, drops in binding affinity (measured as apparent equilibrium association constants) as large as 100-fold were reported (Semenova et al., 2011; Westra et al., 2012). Under pressure from CRISPR-Cas, mobile genetic elements rapidly accumulate such mutations, which allow them to escape CRISPR interference (Deveau et al., 2008; Semenova et al., 2011). In Type I systems spacer acquisition from DNA molecules containing such “escape” protospacers is very strongly stimulated compared to “naïve” adaptation from targets with no matches to crRNA spacers (Datsenko et al., 2012; Fineran et al., 2014; Li et al., 2014; Richter et al., 2014; Westra et al., 2015). This specific version of spacer acquisition is referred to as “primed CRISPR adaptation” (Datsenko et al., 2012). The dramatically different behavior of fully matched (“wild-type,” “wt”) and partially mismatched (“escape,” “esc”) protospacer targets in *E. coli* strains with inducible expression of *cas* genes is shown in Figure 1. As can be seen, the presence of protospacer fully matching the crRNA spacer and harboring a functional PAM decreases plasmid transformation efficiency in cells expressing *cas* genes at least two orders of magnitude compared to control plasmid without protospacer (Figure 1B). A point mutation in PAM-proximal “seed” region of the protospacer (Semenova et al., 2011) restores the transformation efficiency to control level. The experiment can be modified by transforming plasmids in uninduced cells (when *cas* genes are not expressed all plasmids are transformed equally well). Next, transformed cells are allowed to grow in the absence of antibiotic that selects for plasmid maintenance and *cas* gene expression is induced. Upon growth in the presence of inducers, robust spacer adaptation is revealed in cultures harboring a plasmid with mismatched protospacer (Figure 1C, adaptation is detected by PCR, cells that acquired a spacer—and an additional copy of repeat—result in a longer amplification product). No such product is observed in cultures of cells transformed with a plasmid harboring fully matching protospacer with a functional PAM. Analysis of newly acquired spacers shows that most (90–95%) of them are complementary to the strand where the original priming protospacer was located (Datsenko et al., 2012; Swarts et al., 2012; Fineran et al., 2014; Shmakov et al., 2014). This is a hallmark of primed adaptation, since naïve adaptation reveals no such bias: spacers are chosen from both DNA strands with equal efficiency (Yosef et al., 2012). Primed adaptation requires not just Cas1 and Cas2, but also all other components of the effector complex (the subunits of Cascade: Cse1, Cse2, Cas7, Cas5, Cas6e, and the crRNA), and the Cas3 nuclease/helicase (Datsenko et al., 2012). Primed adaptation is highly beneficial to the host, as it leads to specific acquisition of spacers from genetic parasites that “learned” to evade defenses provided by earlier-acquired spacers.

**Figure 1:**
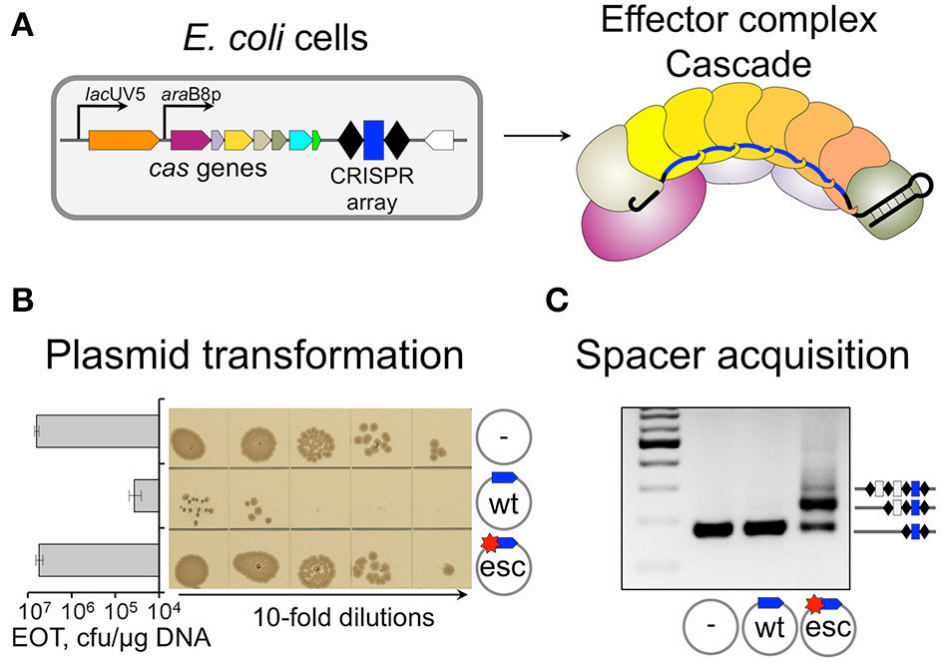
**Biological consequences of a single mismatch between crRNA spacer and plasmid protospacer. (A)** *E. coli* cell with inducible *cas* genes and a CRISPR array containing a single g8 spacer (blue rectangle) and effector complex present in induced cells are schematically shown. Upon induction, effector complex shown on the right is accumulated in the cells. **(B)** Results of transformation of induced cells shown in **(A)** with plasmids carrying fully matching (“wt”) or single-mismatch (“esc” for “escape”) protospacer, or a control vector (transformants grown from serial dilutions of transformation reactions and efficiencies of transformation, EOT) are presented. **(C)** PCR analysis of CRISPR array expansion in induced cultures of cells transformed with indicated plasmids are shown, with amplicons corresponding to expanded and unexpanded arrays indicated.

Primed adaptation clearly relies on specific recognition of partially matching protospacers by Cascade-crRNA effector. The dramatic difference in physiological consequences of recognition of fully and partially matching protospacers (interference vs. primed adaptation) raises a question of whether Cascade-crRNA complexes with two kinds of targets are also different from each other structurally and functionally. Two hypothetical models of primed adaptation based on prior research from several laboratories have been recently put forward. One model summarized by Wright et al. (2016) envisions that effector complex bound to a partially matching target (priming protospacer) asymmetrically recruits Cas3 in the presence of Cas1-Cas2. According to this model, a complex of Cas3, Cas1, and Cas2 then dissociates from the effector complex bound at the priming protospacer and slides along the double-stranded DNA in either direction. As it slides, the Cas3-Cas1-Cas2 complex recognizes PAM sequences located *in cis* with respect to the priming protospacer, excises double-stranded protospacers and channels them for insertion into CRISPR array (Redding et al., 2015; Wright et al., 2016). In the second model, summarized by Amitai and Sorek (2016), binding of the effector complex to a partially matching target causes recruitment of Cas3. The latter directs, through an unspecified mechanism, the Cas1-Cas2 complex to target DNA. In the case of *E. coli* Type I-E system it is proposed that Cas1-Cas2 recognize PAM sequences and excise double-stranded protospacers located at both sides of the bound effector complex (Amitai and Sorek, 2016). Both models envision that naïve adaptation occurs when single-stranded DNA fragments generated by the RecBCD nuclease are bound by Cas1-Cas2, reannealed to form fully or partially double-stranded intermediates, and then processed for insertion into the CRISPR array. In both models Cas3 binding to the effector complex at the fully matching protospacer with a functional PAM causes target destruction without adaptation. In contrast, Cas3 binding to effector complex at partially mismatched priming protospacer leaves DNA bound to the effector complex intact (Wright et al., 2016).

Both models envision that effector complexes bound to fully matching, interference-competent, and partially mismatched, adaptation-competent protospacers are qualitatively different. The structure of Cascade-crRNA complex with a fully matching double-stranded target has been determined (Hayes et al., 2016). The structure reveals an R-loop that is formed as a result of the formation of a perfect 32-bp heteroduplex over the entire length of the crRNA spacer and complementary protospacer. Several studies suggest that the R-loop formation is initiated when the Cse1 subunit of Cascade recognizes the PAM sequence in double-stranded DNA (Sashital et al., 2012; Hochstrasser et al., 2014; Tay et al., 2015; Hayes et al., 2016). One can envision that the presence of mismatches between crRNA spacer and protospacer or imperfect recognition of non-consensus PAM by Cse1 significantly changes the structure, by altering the conformation of protein components, the extent of the R-loop, or both. No structures with mismatched complexes are available at the time of this writing. However, single-molecule analysis has indeed suggested that complexes with mismatched targets may be only partially open (Blosser et al., 2015; Figure 2). Different structures may thus explain the different consequences of effector binding to matched and mismatched protospacers shown in Figures 1B,C.

**Figure 2:**
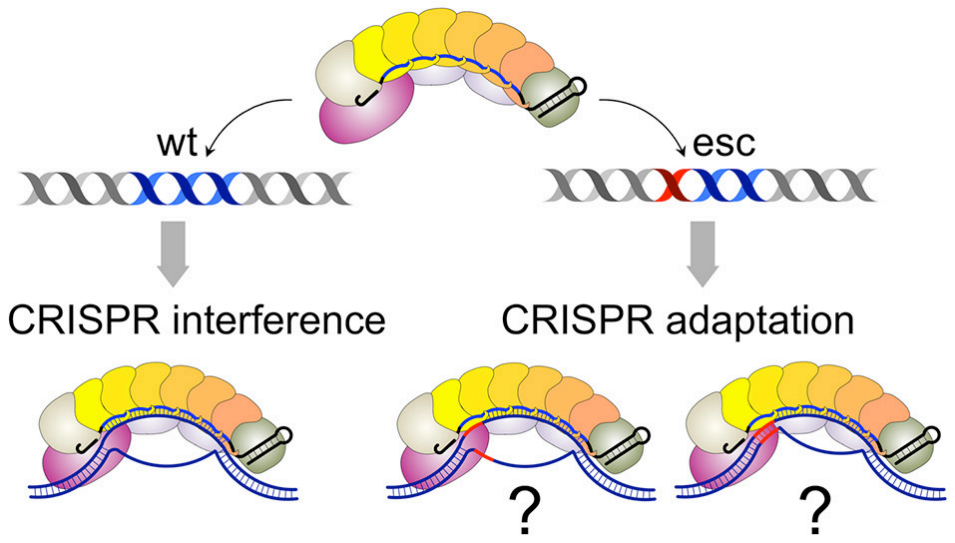
**Cascade-crRNA complexes with matching and mismatched targets**. On matching target, a complex with a fully-opened R-loop is formed (left). Complexes with mismatched targets (mismatch position highlighted in red) may be structurally different (i.e., partially opened, far right) or be the same as matched target complexes.

Recent data from several laboratories (Fineran et al., 2014; Xue et al., 2015) suggest that depending on the spacer-protospacer pair and the kind of mismatch mutation present, a continuum of phenotypes (from 100% interference with no adaptation to efficient adaptation without visible interference) is observed in plasmid transformation interference/primed adaptation experiments similar to that shown in Figures 1B,C. If effector complexes are able to adopt two different functional conformations, the result would suggest that at different targets the relative proportion of such conformations is correspondingly different. However, there is an alternative view that considers interference and primed adaptation as intimately connected processes involving same complexes (Swarts et al., 2012; Semenova et al., 2016). According to this view interference provides substrates for the primed adaptation. Further, the rates of interference (target destruction) and spacer acquisition reactions can't be considered in isolation: copy number maintenance mechanisms of plasmids or phage genome targeted by CRISPR-Cas should also be considered. The latter become very important when one interprets the results of experiments as that shown in Figure 1. DNA harboring fully matching protospacers can be located by Cascade effector rapidly and then destroyed by Cas3. If copy number maintenance mechanisms are not able to keep up with the rate of interference, foreign DNA (and its degradation products) is rapidly purged from cells. If spacer insertion is a slow reaction (compared to the rate of degradation of target DNA by Cas3 and the rate of degradation of Cas3-generated products by cellular nucleases), no CRISPR array expansion is expected to occur in most cells in the culture.

The outcome becomes different when a mismatch mutation in a protospacer decreases the rate of R-loop complex formation, making CRISPR interference less efficient, giving foreign DNA replication/copy maintenance systems a chance to compensate for the pressure from CRISPR interference over extended periods of time and leading to production of phage progeny from infected cell (during phage infection) or continuous maintenance of plasmid in a clonal population of cells arising from a single transformed founder cell. Both the yield of phage particles and the plasmid copy number are expected to decrease compared to values in unprotected cells. The ongoing, “perpetual” interference process in both cases generates a stream of foreign DNA degradation products that are maintained in the cells at a constant steady-state level. These products can be acted upon by adaptation proteins Cas1-Cas2. Preferential adaptation of spacers from foreign DNA becomes a default consequence of specific degradation of foreign (as opposed to host) DNA by the interference machinery. The strand bias observed during primed adaptation seems to suggest that at least initially, Cas3 generated products that are acted upon by Cas1-Cas2 are single-stranded. This would be consistent with the known 3′–5′ polarity of nucleolytic action of Cas3 *in vitro* (Sinkunas et al., 2011). However, the strand bias may also result from Cas1-Cas2 association with Cas3 moving away from the priming site (Richter et al., 2014; Redding et al., 2015) and as such be independent of its exonuclease activity. Alternatively, the mechanism of spacer insertion into CRISPR array may itself be directional and result in orientation bias that is perceived as an apparent strand bias.

To show that an interplay between the rate of target recognition/destruction, spacer adaptation from products generated by target degradation, and target DNA copy number maintenance mechanisms can generate outcomes similar to the ones shown in Figure 1 with just one kind of effector complexes, a simple numerical mathematical model of CRISPR-plasmid dynamics was elaborated. The model assumes that spacers are constantly acquired in CRISPR arrays provided that products of interference generated by effector complex binding to intact plasmids are present. The plasmid copy number *P* in CRISPR-free cells is assumed to be controlled by the logistic dynamics: the growth term is linear in *P* and depends on *replication rate* α and the decay term, manifesting a feedback mechanism, which limits the growth when the plasmid number approaches its target value *P*_0_, is quadratic in *P*:

$$\begin{array}{l} \frac{dP}{dt}=\alpha P\left(1-\frac{P}{P_{0}}\right). \end{array}$$

In the presence of Cas proteins and crRNA recognizing a plasmid protospacer, an additional decay term −β*P* that depends on *CRISPR interference rate* β is introduced:

$$\begin{array}{l} \frac{dP}{dt}=\alpha P\left(1-\frac{P}{P_{0}}\right)-\beta P. \end{array}$$

Thus, ongoing CRISPR interference changes the equilibrium number of plasmids from *P*_0_to (α − β)*P*_0_/α for α > β and to 0 for α < β. The number of plasmid fragments *F* which appear as a result of CRISPR interference is controlled by one gain term, which depends on the efficiency of interference, and two loss terms: the first one describes the intrinsic degradation of such fragments with the rate δ and the second one accounts for acquisition of such fragments as spacers into the bacterial genome with the rate χ

$$\begin{array}{l} \frac{dF}{dt}=C\beta P-\left(\delta +\chi \right)F. \end{array}$$

The conversion factor *C* is the number of protospacers produced from one plasmid once it is recognized by the effector complex and degraded by Cas3. Finally, the number of spacers *S* acquired into an array is given by

$$\begin{array}{l} \frac{dS}{dt}=\chi F. \end{array}$$

To mimic a realistic induction scenario (Figure 1C), we first allow plasmids to reach their CRISPR-free stationary copy number *P*_0_. Then at time *t*_0_ the CRISPR-Cas system is turned on by increasing β(*t*) and χ(*t*) from zero to their stationary values β and χ via a simple transitory regime with rate ρ, which mimics their zero-order production and first-order decay kinetics.

$$\begin{array}{l} \beta (t)=\beta (1-e^{-\rho (t\;-\;t_{0)}}),\\ \text{ χ}(t)=\text{χ}(1-e^{-\rho (t\;-\;t_{0)}}). \end{array}$$

The results for two values of β, β = 0.5α, and β = 1.5α, are shown in Figure 3. As can be seen the model predicts dramatically different outcomes for the two conditions. When β < α (a situation one can expect it for mismatched protospacer target), the plasmid copy number converges to a steady state. The level of degradation products proceeds through and initial increase and then settles on a plateau of its own. The number of spacer acquisition events (and, therefore, cells that underwent adaptation) increases linearly with time, with spacers continuously acquired from a constant pool of plasmid degradation products. Eventually, every cell in the population acquires at least one spacer. The newly acquired spacers are characterized by high level of interference rate β and so the plasmid is lost due to “secondary” interference, however, cells with expanded arrays remain in the population. In contrast, when β > α from the very beginning (as expected for a fully matching spacer-protospacer pair), the plasmid population becomes extinct rapidly, while plasmid degradation products, generated by interference, accumulate sharply and then abruptly decline. Only a few cells in the population acquire spacer during a short time window when plasmid degradation products are present. It follows from the steady-state analysis of the equations above when β = α/2 the maximal rate of spacer acquisition is achieved. When β is zero and CRISPR-Cas system is inactive, spacers are not acquired at all.

**Figure 3:**
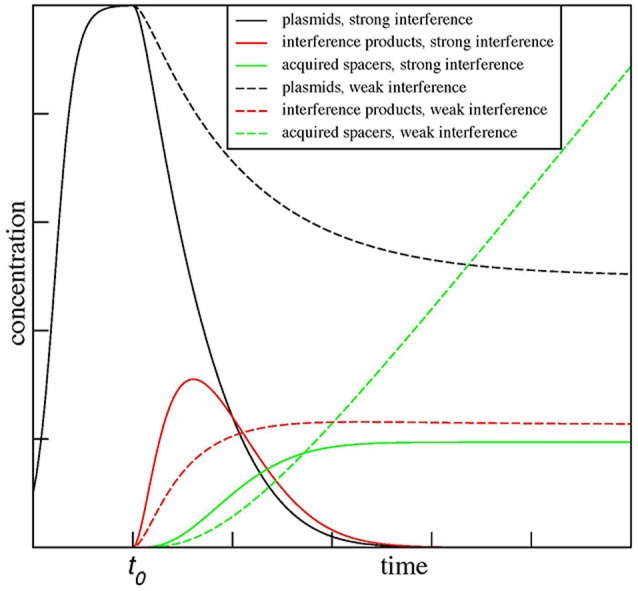
**Kinetic modeling of CRISPR interference and spacer acquisition at different ratios of CRISPR interference and foreign DNA copy number maintenance**. The modeling results show outcomes of CRISPR interference and adaptation depending on the rate of replication of foreign DNA targeted by the CRISPR-Cas system α and the rate of CRISPR interference β. The results for two ratios of these rates, β = 0.5α and β = 1.5α, are shown. When β < α, the plasmid number (dashed black line) converges to a steady state and spacers (dashed green line) are continuously acquired from plasmid degradation products (dashed red line). In contrast, when β > α, the plasmid population dies out (solid black line) and only a few spacers (solid green line) are acquired by the population during a short time when plasmid degradation products (solid red line) are present. The plots are shown for α = *C* = *P*_0_ = δ = 1 and χ = ρ = 0.1.

Our simple mathematical model shows that experimentally observed and seemingly mutually exclusive outcomes of the kind shown in Figure 1 can be achieved with minimal adjustments of parameters and without a requirements for two functionally different kinds of effector complexes at matching and mismatched protospacer targets.

While the model was developed to explain CRISPR-Cas outcomes when targeting plasmids, similar logic can be used to explain the behavior of cells infected with the phage. Though mathematical modeling in this case becomes more complex and will be presented elsewhere, qualitatively, the idea is easy to grasp and is schematically illustrated using the example of M13 phage infection in Figure 4. When cells harboring no CRISPR spacers are infected with the phage the infection of most cells proceeds normally, resulting in phage progeny production (Figure 4A). Some of the infected cells acquire spacers due to the action of the Cas1-Cas2 adaptation complex, which indiscriminately inserts into the CRISPR array fragments of host or viral DNA that have been generated by the replication and/or recombination processes. Cells that acquired host-derived spacers undergo autoimmune death, which could be beneficial for the population of cells by limiting the spread of infection. Cells that acquired phage-derived spacers are able to destroy intracellular viral DNA and survive. They and their progeny can destroy incoming viral DNA (Figure 4B) through effector complex recognition followed by degradation mediated by Cas3. The destruction is rapid and while it generates viral DNA fragments that can be incorporated in the array this does not happened often as these fragments are short-lived. Incorporation of extra spacers can further increase the resistance levels of the host (Brouns et al., 2008; van Houte et al., 2016). Efficient CRISPR interference provides strong selection for phage harboring escape mutations (Figure 4C). Two independent processes unfold in cells infected by the mutant phage. On the one hand, the mutant phage genomes replicate. On the other hand, CRISPR interference also happens, which, however, is not efficient to allow full curing from the phage. As a result, a situation that is similar to the one described in the model of Figure 3 is created. The infected cells will contain substantial steady-state levels of phage DNA destruction products, generated by Cas3, which can be used by the Cas1-Cas2 adaptation complex for array expansion. Unlike the situation shown in Figure 4A, the adaptation process now becomes predominantly targeted to phage DNA. As a result, multiple clones containing various “second-generation” spacers in their arrays appear. Such clones become resistant to both the wild-type and the first generation escape phage, as is shown in Figure 4D.

**Figure 4:**
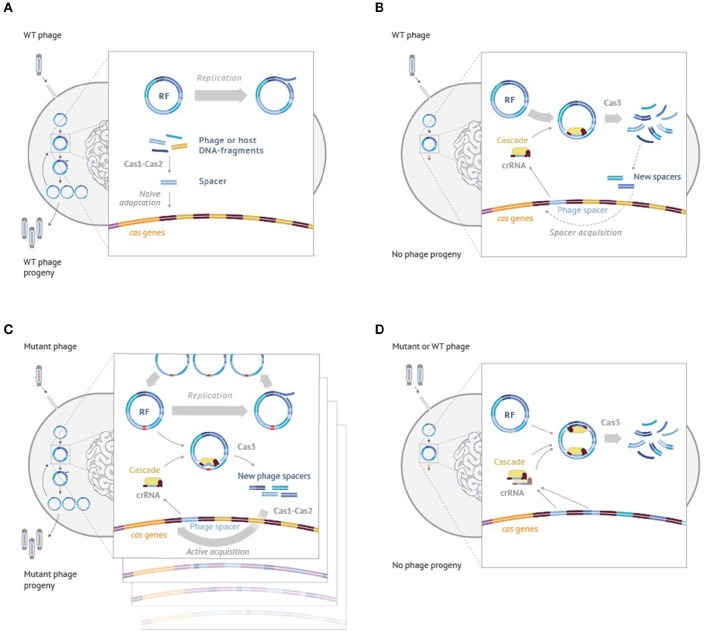
**An interplay between interference and adaptation by Type I-E CRISPR-Cas system and phage DNA replication during the M13 phage infection of *Escherichia coli* cells. (A)** Wild-type M13 phage infects a cell lacking CRISPR spacers matching phage sequences. Phage progeny develops; rare cells undergo naïve adaptation, inserting spacers from the phage or host DNA. **(B)** Wild-type phage infects a cell that had earlier acquired a phage-derived spacer. Phage DNA is efficiently recognized by the effector complex and rapidly degraded by Cas3. No phage progeny is produced. On rare occasions, some short-lived Cas3 degradation products are used by the adaptation machinery causing acquisition of additional phage-derived spacers. **(C)** A mutant phage with an escape mutation infects a cell carrying a partially mismatched phage-derived spacer. Phage DNA is inefficiently recognized by the effector complex. While phage genomes replicate, some are degraded by Cas3. Cas3 degradation products are present throughout the infection and are used by the adaptation machinery causing highly efficient acquisition of additionale phage-derived spacers (primed adaption). **(D)** A wild-type or escape phage infect a cell carrying multiple phage-derived spacers. Phage DNA is recognized by the effector complex at multiple protospacers and degraded by Cas3. No phage progeny is produced. See text for more details.

In our model there is no need for the adaptation complex to slide away from the priming site and excise protospacers along its way as envisioned by existing models of primed adaptation (reviewed and summarized in Amitai and Sorek, 2016; Wright et al., 2016). Instead, spacers are selected from a common pool of independent, freely diffusible substrates, which are generated by the interference machinery and channeled for insertion in CRISPR array by the adaptation enzymes. The low level of naïve adaptation is caused by the rarity of appropriate substrates in the absence of CRISPR interference. The increased level of primed adaptation, and its preference for foreign DNA carrying the priming protospacer is a direct consequence of interference with such DNA by the effector complex and the Cas3 protein. The actual amount of the adaptation substrates (and, therefore, the extent of adaptation) results from an interplay of target protospacer binding by the effector complex, the degradation of DNA molecules bound to effector by Cas3, and the ability of foreign DNA replicons to counter interference by their intrinsic copy maintenance mechanisms. Poor adaptation from fully matching targets is a trivial consequence of their rapid destruction (Semenova et al., 2016).

Our model envisions that R-loop complexes formed on either fully matching or partially mismatched protospacers are either very similar or identical, differing only in times needed for them to form and/or recruit Cas3. Thus, the steady-state amount of such complexes formed on fast-replicating foreign DNA shall also be different. Once formed, open effector complexes recruit Cas3 that proceeds to destroy target DNA, moving progressively away from the protospacer. The lower binding of effector complexes to escape targets slows down the rate of reduction of copy number of plasmids used in standard transformation assays, allowing plasmid copy maintenance mechanism to offset, fully or partially, the CRISPR interference machinery action, essentially creating a condition of perpetual, albeit low-level interference. The products of Cas3 action are then approached by Cas1-Cas2 and channeled for insertion in the CRISPR array. The extent of removal of DNA around the interference site (and stability of Cas3-generated intermediates) may be affected by various non-CRISPR-Cas functions as indeed has been suggested by recent evidence (Ivancic-Bace et al., 2015; Levy et al., 2015). According to this view, the naïve and primed adaptation processes are mechanistically identical and both require only Cas1 and Cas2. The two processes only differ in the way the substrates for Cas1-Cas2 action are generated: through a highly inefficient and random aberrant processes during naïve adaptation or by a highly efficient, directional, and target- and strand-specific Cas3-mediated target DNA destruction during primed adaptation.

The moment a new spacer is acquired, cells raise the ante in this arms race, and the foreign genome is either purged or must respond by the accumulation of mutations in the protospacer and/or PAM corresponding to the newly acquired spacer. The indiscriminative nature of the adaptive response by the CRISPR-Cas system must lead to very rapid diversification of the initial clonal population, since individual cells will be acquiring different spacers from foreign DNA in the course of primed adaptation, as indeed observed in the recent study by van Houte et al. (2016). This in turn should drive corresponding diversification of resident plasmids or infecting viruses that infect such cells and evolve escape mutations or curing of bacterial culture. Mathematical modeling coupled to long-term cultivation experiments will be necessary to study the dynamics of such systems.

## Author contributions

All authors listed, have made substantial, direct and intellectual contribution to the work, and approved it for publication.

### Conflict of interest statement

The authors declare that the research was conducted in the absence of any commercial or financial relationships that could be construed as a potential conflict of interest. The reviewer MD declared a past co-authorship with one of the authors KS to the handling Editor, who ensured that the process met the standards of a fair and objective review.
